# Wear-Smile: A Multidisciplinary Telemedicine-Based Smoking Cessation Program Assisted by Wearable Monitoring Devices for Persons Who Smoke—Protocol for a Randomized Controlled Trial

**DOI:** 10.3390/healthcare14142055

**Published:** 2026-07-09

**Authors:** Maria Pia Di Palo, Massimo Amato, Carmine Vecchione, Michele Ciccarelli, Marina Garofano, Federica Di Spirito, Michele Davide Mignogna, Francesco Corallo, Maria Pagano, Irene Cappadona, Colomba Pessolano, Alessia Nunziante, Alessia Bramanti

**Affiliations:** 1 Department of Medicine, Surgery and Dentistry, University of Salerno, 84081 Baronissi, Italy; mamato@unisa.it (M.A.); cvecchione@unisa.it (C.V.); mciccarelli@unisa.it (M.C.); mgarofano@unisa.it (M.G.); cpessolano@unisa.it (C.P.); alenunziante@unisa.it (A.N.); abramanti@unisa.it (A.B.); 2 Department of Innovative Technologies in Medicine & Dentistry, University “G. d’Annunzio” Chieti-Pescara, 66100 Chieti, Italy; 3 Department of Neuroscience, Reproductive Science and Dentistry, University of Naples Federico II, 80131 Naples, Italy; mignogna@unina.it; 4 IRCCS Centro Neurolesi Bonino-Pulejo, Via Palermo, S.S. 113, C.da Casazza, 98124 Messina, Italyirene.cappadona@irccsme.it (I.C.)

**Keywords:** smoking cessation, smoker, tobacco, telemonitoring, telemedicine, wearable devices, telehealth, oral health, cardiopulmonary health, quality of life

## Abstract

Background: Tobacco smoking remains one of the leading preventable causes of morbidity and mortality worldwide and is associated with cardiovascular, respiratory, oral, and psychological disorders. Although conventional smoking cessation interventions are effective, barriers related to accessibility, adherence, healthcare costs, and continuity of care frequently limit long-term success. Recent advances in technology may offer innovative opportunities to improve multidisciplinary smoking cessation pathways. Methodology: The Wear-Smile project is a non-profit, monocentric, randomized controlled trial registered in the Clinical Trial Protocol Registry and Results System (code No.: NCT07593742), designed to evaluate the effectiveness of a multidisciplinary telemedicine-based smoking cessation program assisted by wearable remote-monitoring devices. Adults who smoke combustible or heated tobacco or electronic nicotine delivery systems will be randomly allocated in a 1:1 ratio to either an experimental group receiving telemedicine-assisted rehabilitation integrated with wearable monitoring devices or a control group receiving standard in-person smoking cessation care. The intervention will include a multidisciplinary behavioral smoking cessation intervention involving cardiology, respiratory, dental, and psychology specialists. Primary outcomes will include Continuous Abstinence Rates and Point Prevalence Abstinence assessed at 6 and 12 months. Secondary outcomes will include cardiopulmonary and oral health parameters, smoking-related variables, health- and oral health-related quality of life (QoL), as well as adherence, usability, acceptability, and satisfaction with the rehabilitation program. Conclusions: The project may provide preliminary evidence regarding the potential feasibility, acceptability, and effectiveness of a multidisciplinary smoking cessation program integrating telemedicine and wearable monitoring technologies in improving abstinence outcomes, patient engagement, continuity of care, and QoL.

## 1. Introduction

The World Health Organization estimates more than 8 million deaths due to direct tobacco smoke and 1.2 million deaths due to second-hand tobacco smoke each year [1].

Tobacco smoking remains one of the leading preventable causes of morbidity and mortality worldwide and represents a major modifiable risk factor for chronic non-communicable diseases [2], including cardiovascular diseases [3], respiratory disorders [4], oral diseases [5,6], and malignant neoplasms [7].

Therefore, smoking cessation programs have a central role in public health interventions, with both short- and long-term improvements for the health of persons who formerly smoked [8].

For instance, improvements in pulmonary function may already be observed within 48 hours after smoking cessation [9]; from a psychological perspective, persons who formerly smoked for at least six weeks registered lower levels of anxiety, stress, and depression symptoms after smoking cessation [10]. Furthermore, within the first three months after quitting, improvements in respiratory performance, walking capacity, and skin appearance have been described, together with a reduction in cardiovascular risk [9]. In the long term, smoking cessation contributes to lowering the incidence of cardiovascular diseases, stroke, respiratory disorders, and malignant neoplasms. Specifically, the risk of oral squamous cell carcinoma has been reported to decrease by approximately 50% after five years of smoking abstinence, while the risk of lung cancer progressively approaches that of persons who never smoked after ten years [11,12].

In this context, different healthcare providers, such as cardiologists, pulmonologists, dentists, and psychologists, should cooperate to achieve physical, social, and psychological health and well-being in persons with nicotine dependence.

Several studies have demonstrated the effectiveness of conventional face-to-face smoking cessation interventions in supporting smoking abstinence, including physician-delivered brief advice [13]. Additional benefits have been observed with more intensive behavioral interventions, such as individual or group-based counseling programs conducted in person [14,15].

Nevertheless, intensive smoking cessation interventions are frequently associated with increased healthcare costs, greater demands on healthcare professionals’ time, and longer waiting periods for persons who smoke seeking access to cessation services [16].

In recent years, advancements in intelligent hardware, software, and big data have led to the widespread adoption of wearable remote-monitoring devices across several healthcare fields, transforming healthcare by supporting a wide range of applications for subjects of all ages and for both physiological and pathological conditions [17,18,19]. These devices are employed to monitor medical parameters, track health-related biomarkers, provide therapeutic functions and remote treatment plan adjustments, and enhance athletic and military performance [17,18,20]. For chronic disease management, wearable devices were used as continuous glucose monitors [21] or to provide real-time electrocardiograms to assist in the treatment of diabetes, cardiovascular diseases, and respiratory conditions [22].

In recent decades, various technological tools have been explored to assess smoking behavior, such as measuring expired carbon monoxide and specific biomarkers. However, these approaches have not enabled the reliable identification of smoking patterns. To address this, a portable monitoring device was developed to detect smoking events, requiring users to smoke exclusively through the device. Nonetheless, compliance issues and the device’s obtrusiveness and bulkiness often led to inaccurate data recording [23].

Taking into consideration the new opportunities offered by wearable remote-monitoring devices for the continuous tracking of health-related parameters, integrating these devices into smoking cessation programs may enhance patient monitoring and support.

Furthermore, combining wearable remote-monitoring devices with an integrated telemedicine platform may facilitate multidisciplinary management by different healthcare providers.

This integrated approach may represent a promising strategy to address smoking, a major public health issue that significantly affects the overall health and well-being of persons who smoke.

The present Wear-Smile study protocol aims to describe the design and methodology of a multidisciplinary smoking cessation program based on telemedicine and wearable remote-monitoring devices for persons who smoke combustible tobacco, non-combustible tobacco products, and other nicotine-containing products.

The Wear-Smile project was designed to investigate whether the integration of wearable monitoring technologies and a telemedicine platform into a multimodal smoking cessation pathway may improve abstinence outcomes, treatment adherence, patient engagement, and health-related outcomes compared with standard in-person smoking cessation care. Furthermore, the protocol aims to evaluate the impact of the intervention on cardiovascular, respiratory, oral health, psychological well-being, and quality of life parameters through a multi-specialistic approach.

## 2. Methodology

### 2.1. Study Design and Setting

The study protocol is designed as a non-profit, monocenter, two-parallel-group (experimental and control groups), non-blinded, controlled randomized trial in persons who smoke combustible tobacco products (conventional cigarettes), heated tobacco products (HTPs), and electronic nicotine delivery systems (ENDSs).

The study protocol adheres to the updated principles of the Declaration of Helsinki and was approved by the local Ethics Committee of Azienda Ospedaliero-Universitaria San Giovanni di Dio e Ruggi d’Aragona (protocol No. 4196/2025 approved on 30 January 2026). The study was also registered in the Clinical Trial Protocol Registry and Results System (https://register.clinicaltrials.gov/, accessed on 11 May 2026) of the United States of America (code No.: NCT07593742; link: https://clinicaltrials.gov/study/NCT07593742, accessed on 26 May 2026).

The study protocol was developed according to the Standard Protocol Items: Recommendations for Interventional Trials (SPIRITs) guidelines [24].

According to the inclusion and exclusion criteria detailed below, eligible participants who voluntarily wish to participate in a smoking cessation program (for physical, social, and/or psychological reasons) and sign a written informed consent will be recruited at the Azienda Ospedaliero-Universitaria San Giovanni di Dio e Ruggi d’Aragona di Salerno, Salerno, Italy.

Before providing the informed consent form to be signed, participants will be informed about the types of data collected within the study and the procedures implemented to ensure data anonymity and confidentiality. They will also receive contact details of the study’s designated coordinator for any queries or concerns, as well as clear instructions regarding their right to withdraw from the Wear-Smile project.

Participants will be randomly distributed equally (in a 1:1 ratio) into the experimental group (EG) and control group (CG). The random allocation sequence will be carried out by the web-based randomization service Research Randomizer (freely accessible online at: https://www.randomizer.org/, accessed on 26 May 2026) by an independent researcher not involved in participant recruitment, eligibility evaluation, or outcome assessment. Allocation concealment will be ensured using sequentially numbered, opaque, sealed envelopes prepared by the independent researcher. The envelopes will be opened sequentially only after the participant has completed the baseline assessment and has been formally enrolled in the study, thereby preventing foreknowledge of treatment allocation and minimizing selection bias.

Due to the nature of the intervention, participants and healthcare providers cannot be blinded to group allocation. Outcome measures will be collected using standardized assessment procedures to minimize assessment bias by independent researchers not involved in participant recruitment, eligibility evaluation, or randomization allocation.

### 2.2. Study Participants

The inclusion criteria will be:

- Person who currently smokes ≥5 cigarettes per day [25] (conventional cigarette or equivalent in nicotine content from HTP or ENDS);
- Adult persons who smoke (≥18 years old);
- Ability and willingness to provide informed consent or presence of a legal representative who consents.

The exclusion criteria will be:

- Persons < 18 years old;
- Pregnant or breastfeeding women;
- Current or past use of pharmacological or behavioral smoking cessation supports [25];
- Concurrent dependence on alcohol or other substances of abuse (excluding nicotine) [25];
- Advanced neurocognitive degenerative disorders;
- Unstable psychiatric conditions (including active suicidal ideation, recent psychiatric hospitalization, and/or changes in psychiatric medication within the past 3 months) [26,27];
- Inability or refusal to provide informed consent;
- Inability to access the integrated telemedicine platform and/or use wearable remote-monitoring devices;
- Participants without access to home medical devices for monitoring cardiopulmonary parameters (blood pressure monitor and pulse oximeter), required to perform the scheduled home assessments.

No restrictions regarding gender, race, nicotine addiction, or motivation to quit smoking will be enforced.

### 2.3. Remote Smoking Cessation Program Assisted by Wearable Monitoring Devices (Experimental Protocol)

A tailored, multimodal behavioral smoking cessation telerehabilitation program assisted by non-invasive wearable remote-monitoring devices will be used for the EG. The program will be integrated with wearable devices and the web-based telemedicine platform BTS TELEREHAB: https://www.btsbioengineering.com/it/products/bts-telerehab/ accessed on 26 May 2026 (BTS Bioengineering S.p.A., Garbagnate Milanese, Italy), accessible via dedicated workstations and/or mobile devices operating on Android or iOS systems.

Participants will receive individualized behavioral and psychological support in accordance with the national clinical guidelines to promote smoking cessation by the Italian Ministry of Health and the National Institute of Public Health (freely available on https://www.salute.gov.it/imgs/C_17_pubblicazioni_832_allegato.pdf, accessed on 12 May 2026), including individual counseling, cognitive behavioral therapy, and motivational interventions aimed at promoting smoking cessation, increasing awareness of the harmful effects of tobacco and nicotine-containing products, identifying smoking triggers, and preventing relapse throughout the rehabilitation process.

All behavioral counseling and support components are standardized between the experimental arm and the control arm; only the delivery modality differs.

The intervention includes periodic counseling sessions focused on smoking cessation, lifestyle modification, pharmacological adherence, and psychological support. These sessions are scheduled at the time of the routine outpatient follow-up visits, which occur with the same frequency in both study arms according to standard clinical practice. The duration of each session is approximately 30 min.

Specialist-specific contributions are as follows:

- Cardiologists: optimization of cardiovascular risk factors, therapeutic management, motivational support for smoking cessation, and counseling on the cardiovascular benefits of quitting smoking.
- Respirologists: assessment and management of respiratory comorbidities, pulmonary function evaluation, support for smoking-related respiratory issues, and counseling on the respiratory benefits of quitting smoking.
- Dentists: evaluation of oral health, management of smoking-related periodontal disease, and counseling on the oral benefits of quitting smoking.
- Psychologists: structured behavioral counseling, motivational interviewing techniques, management of psychological dependence, and emotional support.

All sessions are documented in the electronic health record to ensure traceability, reproducibility, and consistency between the two study arms.

On a voluntary basis, participants allocated to the study group will be asked to use the wearable remote-monitoring devices and record physiological parameters twice a day, for a maximum duration of the study of 12 months.

Following enrollment, the EG will receive training on the use of the integrated telemedicine platform and the wearable monitoring devices after the calibration of the devices.

The wearable devices will record participants’ cardiopulmonary parameters (heart rate, blood pressure, respiratory rate, and oxygen saturation) on the integrated telemedicine platform. In this way, the participant’s medical parameters will be automatically recorded through the wearable device and automatically transferred to the telemedicine platform.

Participants will also complete a paper-based smoker diary developed by the Italian Ministry of Health and the National Institute of Public Health (freely available on https://smettodifumare.iss.it/documents/d/guest/guida-smetto-di-fumare-a5-v07, accessed on 12 May 2026), recording the daily number and frequency of cigarettes or nicotine-containing products used.

Adherence to wearable device utilization will be automatically tracked through the telemedicine platform by recording the timing and frequency of device use.

Participants in the EG will also self-report oral health-related parameters, including gingival bleeding during daily oral hygiene procedures performed at least twice a day throughout the rehabilitation program.

All detected smoking events and recorded parameters will be automatically transmitted to the telemedicine platform. In this way, the smoker’s diary will be automatically compiled through the wearable device recordings.

Finally, the integrated telemedicine platform and remote wearable monitoring devices will send push notifications to remind patients to take prescribed medications (particularly for fragile patients and those with comorbidities) at scheduled times.

The telemedicine platform and wearable devices will also periodically send text messages, photos, and videos to support smoking cessation.

Healthcare providers involved in the multidisciplinary smoking cessation program, including dental, cardiologic, pneumological, and psychological specialists, will be able to remotely monitor participants throughout the telerehabilitation program. When clinically necessary, treatment modifications may be proposed and shared among specialists and participants.

At any time, the patient will be able to request and schedule teleconsultation sessions via videoconference with the selected healthcare specialist through the integrated telemedicine platform, and vice versa. An in-person clinical evaluation should be scheduled if deemed necessary by the clinician.

The integrated telemedicine platform and wearable monitoring system will comply with applicable data privacy and security regulations.

Both the integrated telemedicine platform and wearable remote-monitoring devices will ensure compliance with data privacy and protection standards.

#### 2.3.1. Web-Based Telemedicine Platform

The BTS TELEREHAB platform (https://www.btsbioengineering.com/it/products/bts-telerehab/ accessed on 26 May 2026) is a web-based telemedicine system specifically designed to support remote multidisciplinary care, telemonitoring, and video consultations between healthcare professionals and participants. The platform is accessible through dedicated workstations and patient interfaces and integrates wearable medical devices for the automatic acquisition and transmission of physiological data, allowing real-time remote monitoring and review by the multidisciplinary clinical team. Data acquired from wearable devices and patient-reported outcomes will be automatically uploaded to the cloud-based platform.

To ensure patient privacy and data protection, all personal and clinical data collected through the telemedicine platform will be processed in accordance with the General Data Protection Regulation (GDPR; Regulation (EU) 2016/679). Participants will be identified using unique alphanumeric study codes, and only pseudonymized data will be used for study analyses.

Access to the platform will be restricted to authorized users according to their assigned roles, and the platform will implement appropriate technical and organizational measures to ensure the secure processing, storage, and transmission of health data in compliance with applicable data protection regulations.

#### 2.3.2. Wearable Remote-Monitoring Devices

The wearable remote-monitoring devices integrated with the BTS TELEREHAB platform are medical-grade, non-invasive tools, including wireless inertial sensors (e.g., G-SENSOR) and pulse oximeters (BTS Bioengineering S.p.A., Garbagnate Milanese, Italy). They collect the following physiological parameters: heart rate, blood pressure, respiratory rate, and peripheral oxygen saturation. Measurements will be collected twice daily for up to 12 months.

Data are acquired by the wearable devices and transmitted in real-time via a secure Bluetooth connection to the patient’s mini-PC Brain unit, which then forwards them to the cloud-based BTS TELEREHAB platform (https://www.btsbioengineering.com/it/products/bts-telerehab/ accessed on 26 May 2026) through an Internet connection (provided there is access to Wi-Fi or a portable hotspot). Data transmission uses encrypted protocols compliant with GDPR.

The system is designed for home and clinical use and has been validated in rehabilitation and telemedicine settings, as described in the BTS TELEREHAB (BTS Bioengineering S.p.A., Garbagnate Milanese, Italy) manuals.

Adherence to device use and data quality will be automatically monitored by the platform.

### 2.4. Standard Smoking Cessation Program (Control Protocol)

A tailored, multimodal behavioral smoking cessation rehabilitation program delivered exclusively through in-person care will be used for the CG. The behavioral and psychological interventions provided to the CG will be equivalent to those delivered to the EG (described in Section 2.3), differing only in the mode of delivery, namely, face-to-face assistance rather than telemedicine-based rehabilitation.

The behavioral and psychological interventions will be provided during follow-up visits in order to address behavioral difficulties, maintain motivation, and support long-term abstinence.

For home monitoring of cardiopulmonary parameters, portable medical devices already available in participants’ possession, such as sphygmomanometers and pulse oximeters, will be used to manually record heart rate, blood pressure, respiratory rate, and oxygen saturation twice a day for up to 12 months. Unlike the EG, physiological parameters in the CG will not be automatically transmitted to a telemedicine platform, and no wearable remote-monitoring devices will be used. No participant will be required to independently purchase portable medical devices in order to participate in the study, according to the aforementioned exclusion criteria for the CG.

Similar to the EG, participants allocated to the CG will complete a paper-based smoker diary developed by the Italian Ministry of Health and the National Institute of Public Health (freely available on https://smettodifumare.iss.it/documents/d/guest/guida-smetto-di-fumare-a5-v07, accessed on 12 May 2026), recording the daily number and frequency of cigarettes or nicotine-containing products used.

Participants in the CG will also self-report oral health-related parameters, including gingival bleeding during daily oral hygiene procedures performed at least twice a day throughout the rehabilitation program.

Healthcare providers involved in the multidisciplinary smoking cessation program, including dental, cardiologic, pneumological, and psychological specialists, will monitor participants exclusively through scheduled in-person visits according to the rehabilitation program and participants’ clinical needs. Additional in-person specialist evaluations may be scheduled whenever considered clinically necessary.

### 2.5. Outcomes and Data Collection

#### 2.5.1. Primary Outcome

The primary outcome of the study will be the effectiveness of the program on smoking cessation, assessed through Continuous Abstinence Rates (CARs) and Point Prevalence Abstinence (PPA).

CARs will be defined as sustained smoking abstinence throughout the follow-up period without relapse [28]. CARs will be calculated as the proportion of participants who maintain continuous smoking abstinence at the 6-month and 12-month follow-up assessments without relapse during the observation period.

PPA will be defined as abstinence from smoking at the time of assessment, regardless of any previous smoking relapses during follow-up [28]. PPA will be calculated as the proportion of participants reporting abstinence from smoking at the 6-month and 12-month follow-up assessments, regardless of previous smoking relapses during follow-up.

Both CARs and PPA will be assessed using self-reported smoking status collected through the smoker diary and will be computed 6 and 12 months after baseline [28]. Smoking abstinence outcomes will be assessed through self-reported smoking status, consistent with the telemedicine-based design of the Wear-Smile project.

No biochemical verification of smoking abstinence (e.g., exhaled carbon monoxide or cotinine testing) will be performed.

The timing at 6 and 12 months to assess smoking behavior status was established because a 6-month abstinence period was considered indicative of long-term smoking cessation, as it allows for a more reliable estimation of the proportion of participants who will maintain abstinence even in the subsequent years [8]. A permanent effect of smoking cessation treatment was estimated to be 50% of the Continuous Abstinence Rate at 6 months [8].

#### 2.5.2. Secondary Outcomes

Secondary outcomes will include the longitudinal assessment of cardiopulmonary parameters, oral health conditions, smoking-related outcomes, health-related quality of life, oral health-related quality of life, and participants’ adherence, usability, acceptability, and patient and healthcare satisfaction with the rehabilitation program.

- Cardiopulmonary parameters will include heart rate (beats per minute, bpm), blood pressure (mmHg), respiratory rate (breaths per minute), and oxygen saturation (SpO_2_, %). Heart rate, blood pressure, respiratory rate, and oxygen saturation will be measured twice daily throughout the 12-month study period. In the EG, these parameters will be automatically recorded twice daily through wearable remote-monitoring devices and transmitted to the integrated telemedicine platform. In the CG, the same parameters will be manually self-recorded twice daily using portable medical devices (sphygmomanometers and pulse oximeters) already available in participants’ possession. In addition, in-person clinical cardiology follow-up evaluations will be performed at baseline, 6 months, and 12 months by cardiology specialists for both EG and CG.
- Oral health outcomes will include probing depth (PD, mm) [29], full-mouth bleeding index (FMBI, percentage of bleeding sites) [30], and full-mouth plaque index (FMPI, percentage of plaque-positive sites) [31]. These clinical periodontal parameters will be assessed by calibrated dental specialists during standardized in-person periodontal examinations at baseline, 6 months, and 12 months. Periodontal examinations will be performed using a manual UNC-15 periodontal probe, and the periodontal parameters will be measured at six sites per tooth.

In addition, self-reported gingival bleeding during toothbrushing will be self-reported twice daily throughout the study period.

- Smoking-related secondary outcomes will include the daily number of cigarettes smoked or equivalent nicotine-containing products used, continuously recorded throughout the study period using the paper-based smoker diary. Nicotine dependence severity will be assessed at baseline, 6 months, and 12 months using the Fagerström Test for Nicotine Dependence (FTND) [32]. Smoking cessation motivation will be evaluated at the same time points using the motivational test developed by the Italian National Institute of Public Health (freely available online on https://smettodifumare.iss.it/documents/d/guest/guida-smetto-di-fumare-a5-v07, accessed on 12 May 2026).
- Patient-reported outcomes will include health-related quality of life assessed using the Short Form Health Survey (SF-36) [33], oral health-related quality of life assessed using the Oral Health Impact Profile-14 (OHIP-14) [34], and self-perceived periodontal health assessed using the gingival self-assessment questionnaire developed by the Italian Society of Periodontology and Implantology and the European Federation of Periodontology (freely available online on https://www.gengive.org/test-di-autovalutazione-delle-gengive/, accessed on 12 May 2026). All questionnaires will be administered at baseline, 6 months, and 12 months.
- Technology- and rehabilitation-related outcomes will be assessed at 6 and 12 months and will include usability of the telemedicine platform evaluated through the System Usability Scale (SUS) [35], treatment acceptability and adherence evaluated through the Treatment Acceptability/Adherence Scale (TAAS) [35], and patient satisfaction evaluated through the Client Satisfaction Questionnaire (CSQ-8) [35].
- Adherence to the rehabilitation program and compliance with wearable device utilization will be assessed at baseline, 6 months, and 12 months. In the EG, adherence to wearable device use will be automatically monitored through the telemedicine platform by recording the timing and frequency of device utilization. In the CG, adherence to the rehabilitation program will be evaluated during scheduled in-person follow-up visits.

### 2.6. Statistical Analysis

An a priori sample size estimation was performed using G*Power software version 3.1.9.7 [36]. The calculation was based on the primary outcome (CAR), assuming a one-tailed test, a type I error (α) of 0.05, a statistical power (1−β) of 0.80, and a medium expected effect size. Based on these assumptions, the estimated sample size is 102 participants (51 per group).

The data collected will be pseudonymized (a technique that modifies and masks personal and sensitive data of a natural person to prevent direct or easy attribution to the individual) and will be used exclusively for the purposes of the Wear-Smile study project.

Statistical analyses will be performed according to the intention-to-treat (ITT) principle. Per-protocol analyses will additionally be conducted as sensitivity analyses.

The statistical analysis will be applied to analyze continuous and categorical variables, with particular attention to the handling of missing data and the analysis of both intra- and inter-subject variability.

To enhance data interpretation and understanding of distributions, graphical representations such as histograms, boxplots conditioned on the target variable, and scatter plots will be employed.

To minimize the risk of bias due to missing data, a missing data analysis will be performed to assess whether data are Missing Completely at Random (MCAR) or dependent on other variables. If missing data exceeds 5%, it will be handled using multiple imputation techniques based on appropriate statistical models, thereby reducing the risk of bias.

The adoption of these methodologies will strengthen the scientific evidence regarding the effectiveness of telerehabilitation for smoking cessation, optimize result interpretation, and provide valuable insights for future clinical implementations and public health policies.

A sensitivity analysis will be conducted to compare results between the original datasets and the imputed datasets.

Statistical analyses will be performed using SPSS version 28.0, R version 4.2, or STATA version 17.0. The level of statistical significance will be set at *p* < 0.05 for all tests, with Bonferroni correction applied for multiple comparisons to control the risk of Type I error. Effect sizes and 95% confidence intervals (95% CI) will also be reported whenever appropriate.

Before performing inferential analyses, an Exploratory Data Analysis (EDA) will be conducted to describe the characteristics of the sample and to identify any anomalies or patterns in the data.

Continuous variables will be presented as mean and standard deviation (SD) for normally distributed data and as median and interquartile range (IQR) for non-normally distributed data. Categorical variables will be reported as frequencies and percentages. The Shapiro–Wilk test will be used to assess the normality of distributions and determine the appropriate use of parametric or non-parametric tests.

Longitudinal changes in primary and secondary outcomes over time (baseline, 6 months, and 12 months) will additionally be analyzed using mixed-effects regression models accounting for repeated measurements within participants.

Comparisons between the intervention group (telerehabilitation using the integrated telemedicine platform and wearable remote-monitoring devices) and the control group (standard multimodal behavioral-psychological treatment without telemedicine or wearable devices) will be performed using Student’s t-test for normally distributed continuous variables and the Mann–Whitney U test for non-normally distributed variables.

Within-group longitudinal comparisons across follow-up assessments will be performed using paired Student’s t-tests or Wilcoxon signed-rank tests, as appropriate according to data distribution.

Comparisons between categorical variables, including smoking cessation status (abstinent/non-abstinent), will be performed using the Chi-square test or Fisher’s exact test when appropriate.

Associations between the intervention and both primary and secondary outcomes will be explored using Pearson’s correlation coefficient for normally distributed variables and Spearman’s rank correlation coefficient for non-normally distributed variables.

To control for potential confounding factors (e.g., age, gender, comorbidities), multivariate linear regression models will be applied. In addition, multiple logistic regression will be used to identify predictors of successful smoking cessation. The dependent variable will be smoking cessation status (yes/no), and the independent variables will include type of nicotine use (combustible tobacco vs. HTP vs. ENDS), age, gender, motivation, dependence severity, and comorbidities.

Pre-specified subgroup analyses will be performed according to the type of nicotine-containing product used (combustible tobacco cigarettes, heated tobacco products, and electronic nicotine delivery systems), dependence severity, and motivation to quit. In addition, the type of nicotine-containing product will be included as a covariate in multivariable regression models to account for its potential confounding effect on smoking cessation outcomes.

### 2.7. Trial Status

At the time of study protocol submission, the Wear-Smile project is in the pre-recruitment phase, and participant enrollment has not yet started.

The SPIRIT 2025 participant timeline template [24] was compiled in Table 1 to outline the schedule of enrollment, interventions, and assessments.

## 3. Discussion

The present study protocol describes the design and methodology of the Wear-Smile project, a multidisciplinary smoking cessation program integrating telemedicine and wearable remote-monitoring technologies into a rehabilitation pathway for persons who smoke combustible tobacco, heated tobacco products, and electronic nicotine delivery systems.

Tobacco smoking remains one of the leading preventable causes of morbidity and mortality worldwide and represents a major modifiable risk factor for chronic non-communicable diseases, including cardiovascular diseases, respiratory disorders, oral diseases, and malignant neoplasms [2,37].

In addition to physical consequences, smoking behavior is closely interconnected with psychological well-being, anxiety, depression, and social determinants of health [38]. Consequently, smoking cessation should not be considered exclusively a behavioral intervention but rather a comprehensive multidimensional healthcare strategy requiring collaboration among different healthcare providers.

The Wear-Smile project was conceived within this multidimensional framework. The integration of cardiology, pulmonary, dental, and psychological expertise may contribute to a more holistic management of persons who smoke, particularly in patients with multiple comorbidities due to tobacco. The concept of frailty is increasingly relevant in contemporary healthcare systems due to population aging and the growing prevalence of chronic diseases associated with modifiable risk factors such as tobacco use [39,40]. Fragile individuals often require continuous monitoring, multidisciplinary care, frequent healthcare access, and long-term therapeutic adherence, representing a major challenge for healthcare sustainability [41,42]. In this context, telemedicine and wearable monitoring technologies may offer valuable opportunities to improve continuity of care, patient engagement, remote monitoring, and healthcare accessibility.

Recent advances in wearable technologies and integrated telemedicine systems have expanded the potential applications of remote healthcare across several medical fields [43,44]. Wearable monitoring devices may facilitate continuous longitudinal assessment of physiological parameters while simultaneously promoting patient empowerment and self-awareness regarding health behaviors [45]. In smoking cessation programs, these technologies may additionally improve adherence to rehabilitation pathways [45].

Despite the increasing diffusion of telemedicine interventions for smoking cessation, many available interventions are limited to isolated digital tools, such as mobile applications or messaging systems, without implementing a truly integrated multidisciplinary rehabilitation model [16,46]. The Wear-Smile project was designed to address these limitations by combining wearable physiological monitoring, telemedicine-assisted multidisciplinary care, and longitudinal assessment of cardiopulmonary, oral, psychological, and quality-of-life outcomes.

Although telemedicine and wearable technologies offer important opportunities to improve access to multidisciplinary smoking cessation services, their implementation should also be considered from a health equity perspective. Digital health interventions have the potential to reduce geographical barriers and improve continuity of care, particularly for individuals living in remote or underserved areas [47,48,49]. However, these benefits may not be equally distributed across the population. Differences in socioeconomic status, educational level, digital literacy, internet connectivity, and access to technological resources may influence both participation in telemedicine-based interventions and long-term engagement with digital health solutions. Consequently, individuals with fewer economic or technological resources may be underrepresented in digital healthcare programs, potentially limiting the generalizability of study findings and widening existing health disparities. [47,48,49]. In the present study, participants allocated to the control group will be required to have access to home monitoring devices for the scheduled home assessments. This requirement may limit participation among individuals with fewer economic resources and should therefore be considered when interpreting the external validity and generalizability of the findings. Nevertheless, identifying these practical challenges may provide valuable information for the future development of more equitable telemedicine-based smoking cessation programs. Future large-scale studies should aim to reduce barriers to participation by ensuring broader access to monitoring technologies and digital health resources, thereby promoting more equitable implementation of multidisciplinary telemedicine interventions [48,49].

Another relevant strength of the present protocol is the inclusion of persons who smoke different nicotine-containing products, including combustible tobacco, heated tobacco products, and electronic nicotine delivery systems. This aspect may improve the external validity and generalizability of the findings, considering the increasing global diffusion of alternative nicotine delivery products and the ongoing debate regarding their long-term health effects and role within smoking cessation pathways [50].

The present protocol additionally aims to investigate patient-centered outcomes, including usability, treatment acceptability, adherence, and satisfaction with telemedicine-assisted rehabilitation. These aspects are important because two previous umbrella reviews focusing on the use of technology in smoking cessation programs have highlighted a marked lack of data on acceptability and satisfaction [16,51]. Furthermore, low adherence and limited accessibility have been frequently reported as barriers to traditional smoking cessation services [52]. Telemedicine-based interventions may reduce geographical, organizational, and time-related barriers while improving flexibility and continuity of specialist support [53].

Nevertheless, some limitations of the study should be acknowledged. Due to the nature of the intervention, blinding of participants and healthcare providers is not feasible, potentially increasing the risk of performance bias. Smoking abstinence outcomes will primarily rely on self-reported measures, which may introduce reporting bias. In addition, monocentric design may limit the generalizability of the results to different healthcare settings and populations. Potential variability in digital literacy and technological confidence among participants may also influence adherence to wearable device utilization and engagement with telemedicine platforms. Differences in participants’ acceptance of digital health technologies and their long-term engagement with wearable devices may further affect adherence to the intervention and contribute to attrition bias.

To mitigate these risks related to technology adoption and participant engagement, participants allocated to the intervention group will receive standardized training on the use of the telemedicine platform and wearable devices, together with ongoing technical support throughout the study. Furthermore, adherence to the intervention and device utilization will be monitored during follow-up visits to identify potential barriers to engagement. Differential engagement between the intervention and control groups may also represent a potential source of bias and will be considered when interpreting the study findings and conducting sensitivity analyses.

Despite these limitations, the Wear-Smile project may provide important preliminary evidence regarding the feasibility, acceptability, and potential effectiveness of integrating telemedicine and wearable remote-monitoring devices into multidisciplinary smoking cessation programs. If demonstrated to be effective, this model may contribute to improving smoking abstinence rates, reducing smoking-related morbidity, enhancing quality of life, and optimizing healthcare resource utilization. Moreover, the implementation of integrated telemedicine pathways may facilitate stronger collaboration networks among healthcare professionals involved in smoking cessation and chronic disease prevention.

Future multicenter studies with longer follow-up periods will be necessary to further validate the findings and evaluate the long-term sustainability and cost-effectiveness of telemedicine-assisted smoking cessation interventions in different clinical and healthcare contexts.

## 4. Conclusions

The Wear-Smile project introduces a multidisciplinary smoking cessation rehabilitation program integrating telemedicine and wearable remote-monitoring technologies for persons who smoke combustible tobacco, heated tobacco products, and electronic nicotine delivery systems.

The protocol was designed to address the multifactorial nature of tobacco dependence through the collaboration of cardiologists, pulmonologists, dentists, and psychological specialists to improve smoking cessation outcomes and promote a more patient-centered approach to care.

The integration of wearable monitoring devices and an interactive telemedicine platform may enhance continuity of care, remote monitoring, patient engagement, and adherence to rehabilitation programs while reducing organizational and geographical barriers frequently associated with conventional smoking cessation services. Furthermore, the longitudinal evaluation of cardiopulmonary, oral health, psychological, and quality-of-life outcomes may contribute to a broader understanding of the multidimensional effects of smoking cessation interventions.

The Wear-Smile protocol may provide important preliminary evidence regarding the feasibility and clinical applicability of telemedicine-assisted smoking cessation pathways. If demonstrated to be effective, this integrated model may contribute to improving long-term smoking abstinence, reducing smoking-related morbidity, optimizing healthcare resource utilization, and strengthening multidisciplinary collaboration in preventive healthcare.

## Figures and Tables

**Table 1:** SPIRIT 2025 [24] participant timeline: summary of the Wear-Smile project timepoints of the enrollment, intervention/comparator, and assessments.

TRIAL PERIOD						
	Enrollment		Post-randomization			Close-out
TIMEPOINT	−t_i_ to 0	0		t_1=6 months_		t_2=12 months_
ENROLLMENT:	X					
Eligibility screen	X					
Informed consent	X					
Allocation and Randomization		X				
INTERVENTION/COMPARATOR:						
Smoking cessation telerehabilitation program		X				
In-person smoking cessation program		X				
ASSESSMENTS:						
Demographics variables		X				
Smoking behavior		X	X	X	X	X
Continuous Abstinence Rate (CAR)		X		X		X
Point Prevalence Abstinence (PPA)		X		X		X
Fagerström Test for Nicotine Dependence (FTND)		X		X		X
Heart rate (beats per minute, bpm)		X	X	X	X	X
Blood pressure (millimeters of mercury, mmHg)		X	X	X	X	X
Respiratory rate (breaths/minute)		X	X	X	X	X
Oxygen saturation (SpO_2_, %)		X	X	X	X	X
Probing depth (millimeters, mm)		X		X		X
Full-mouth bleeding index (%)		X		X		X
Full-mouth bleeding index (%)		X		X		X
Oral Health Impact Profile (OHIP-14)		X		X		X
Short form health survey (SF-36)		X		X		X
Usability				X		X
Acceptability				X		X
Satisfaction				X		X

## Data Availability

No new data were created or analyzed in this study.

## References

[1] World Health Organization (WHO) Tobacco. https://www.who.int/health-topics/tobacco.

[2] World Health Organization (WHO) Tobacco and Noncommunicable Diseases. https://www.who.int/europe/publications/m/item/tobacco-and-noncommunicable-diseases.

[3] Kondo T., Nakano Y., Adachi S., Murohara T. (2019). Effects of Tobacco Smoking on Cardiovascular Disease. Circ. J..

[4] Saracen A. (2016). Cigarette Smoking and Respiratory System Diseases in Adolescents.

[5] Larsson S.C., Burgess S. (2022). Appraising the causal role of smoking in multiple diseases: A systematic review and meta-analysis of Mendelian randomization studies. eBioMedicine.

[6] Sangiovanni G., Giordano F., Acerra A., Chiacchio A., Valletta A. (2023). Surgical Extrusion: State of the Art and Literature Review. J. Osseointegration.

[7] Ford P.J., Rich A.M. (2021). Tobacco Use and Oral Health. Addiction.

[8] West R. (2007). The clinical significance of ‘small’ effects of smoking cessation treatments. Addiction.

[9] World Health Organization (WHO) WHO Clinical Treatment Guideline for Tobacco Cessation in Adults. https://www.who.int/publications/b/74755.

[10] Taylor G.M., Lindson N., Farley A., Leinberger-Jabari A., Sawyer K., Naudé R.T.W., Theodoulou A., King N., Burke C., Aveyard P. (2021). Smoking cessation for improving mental health. Cochrane Database Syst. Rev..

[11] The Oral Cancer Foundation Tobacco Cessation. https://oralcancerfoundation.org/understanding/tobacco/tobacco-cessation/.

[12] Di Spirito F., Amato A., Romano A., Dipalma G., Xhajanka E., Baroni A., Serpico R., Inchingolo F., Contaldo M. (2022). Analysis of Risk Factors of Oral Cancer and Periodontitis from a Sex- and Gender-Related Perspective: Gender Dentistry. Appl. Sci..

[13] Stead L.F., Bergson G., Lancaster T. (2008). Physician Advice for Smoking Cessation. Cochrane Database Syst. Rev..

[14] Stead L.F., Carroll A.J., Lancaster T. (2017). Group behaviour therapy programmes for smoking cessation. Cochrane Database Syst. Rev..

[15] Lancaster T., Stead L.F. (2017). Individual Behavioural Counselling for Smoking Cessation. Cochrane Database Syst. Rev..

[16] Di Spirito F., Di Palo M.P., Garofano M., Del Sorbo R., Allegretti G., Rizki I., Bartolomeo M., Giordano M., Amato M., Bramanti A. (2025). Effectiveness and Adherence of Pharmacological vs. Non-Pharmacological Technology-Supported Smoking Cessation Interventions: An Umbrella Review. Healthcare.

[17] Chen X., Kim D.-H., Lu N. (2024). Introduction: Wearable Devices. Chem. Rev..

[18] Lu L., Zhang J., Xie Y., Gao F., Xu S., Wu X., Ye Z. (2020). Wearable Health Devices in Health Care: Narrative Systematic Review. JMIR mHealth uHealth.

[19] Pisano M., Bramanti A., Di Spirito F., Di Palo M.P., De Benedetto G., Amato A., Amato M. (2025). Reviewing Mobile Dental Apps for Children with Cognitive and Physical Impairments and Ideating an App Tailored to Special Healthcare Needs. J. Clin. Med..

[20] Cheng Y., Wang K., Xu H., Li T., Jin Q., Cui D. (2021). Recent developments in sensors for wearable device applications. Anal. Bioanal. Chem..

[21] Luo J., Zhang K., Xu Y., Tao Y., Zhang Q. (2021). Effectiveness of Wearable Device-based Intervention on Glycemic Control in Patients with Type 2 Diabetes: A System Review and Meta-Analysis. J. Med. Syst..

[22] Park Y.-S., An C.-S., Lim C.-G. (2021). Effects of a Rehabilitation Program Using a Wearable Device on the Upper Limb Function, Performance of Activities of Daily Living, and Rehabilitation Participation in Patients with Acute Stroke. Int. J. Environ. Res. Public Health.

[23] Imtiaz M.H., Ramos-Garcia R.I., Wattal S., Tiffany S., Sazonov E. (2019). Wearable Sensors for Monitoring of Cigarette Smoking in Free-Living: A Systematic Review. Sensors.

[24] Chan A.-W., Boutron I., Hopewell S., Moher D., Schulz K.F., Collins G.S., Tunn R., Aggarwal R., Berkwits M., A Berlin J. (2025). SPIRIT 2025 statement: Updated guideline for protocols of randomised trials. BMJ.

[25] Webb J., Peerbux S., Ang A., Siddiqui S., Sherwani Y., Ahmed M., MacRae H., Puri H., Majeed A., Glasner S. (2022). Long-Term Effectiveness of a Clinician-Assisted Digital Cognitive Behavioral Therapy Intervention for Smoking Cessation: Secondary Outcomes From a Randomized Controlled Trial. Nicotine Tob. Res..

[26] Johnson A.L., Kaye J., Baker T.B., Fiore M.C., Cook J.W., Piper M.E. (2020). Psychiatric comorbidities in a comparative effectiveness smoking cessation trial: Relations with cessation success, treatment response, and relapse risk factors. Drug Alcohol. Depend..

[27] American Psychiatric Association (2020). The American Psychiatric Association Practice Guideline for the Treatment of Patients with Schizophrenia.

[28] Hughes J., Keely J., Niaura R., Ossip-Klein D., Richmond R., Swan G. (2003). Measures of Abstinence in Clinical Trials: Issues and Recommendations. Nicotine Tob. Res..

[29] Glavind L., Löe H. (1967). Errors in the clinical assessment of periodontal destruction. J. Periodontal Res..

[30] Lang N.P., Joss A., Tonetti M.S. (1996). Monitoring disease during supportive periodontal treatment by bleeding on probing. Periodontology 2000.

[31] O’LEary T.J., Drake R.B., Naylor J.E. (1972). The Plaque Control Record. J. Periodontol..

[32] Fagerstrom K.O., Kunze M., Schoberberger R., Breslau N., Hughes J.R., Hurt R.D., Puska P., Ramstrom L., Zatonski W. (1996). Nicotine dependence versus smoking prevalence: Comparisons among countries and categories of smokers. Tob. Control..

[33] Apolone G., Mosconi P. (1998). The Italian SF-36 Health Survey. J. Clin. Epidemiol..

[34] Franchignoni M., Giordano A., Brigatti E., Migliario M., Levrini L., Ferriero G. (2010). The Psychometric Property of the Italian Version of the Short-Form Oral Health Impact Profile (OHIP-14). G. Ital. Med. Lav. Ergon..

[35] Hajesmaeel-Gohari S., Khordastan F., Fatehi F., Samzadeh H., Bahaadinbeigy K. (2022). The most used questionnaires for evaluating satisfaction, usability, acceptance, and quality outcomes of mobile health. BMC Med. Inform. Decis. Mak..

[36] Faul F., Erdfelder E., Buchner A., Lang A.-G. (2009). Statistical power analyses using G*Power 3.1: Tests for correlation and regression analyses. Behav. Res. Methods.

[37] Sisalli L., Giordano F., Chiacchio A., Acerra A., Caggiano M. (2023). Medication-Related Osteonecrosis of the Jaw: A Case Report of an Unusual Side Effect of Adalimumab. Case Rep. Dent..

[38] Fluharty M., Taylor A.E., Grabski M., Munafò M.R. (2016). The Association of Cigarette Smoking with Depression and Anxiety: A Systematic Review. Nicotine Tob. Res..

[39] Li W., Xue X., Li D., Pan Y., Xie M., Zhang Y., Zheng W., Zhang M. (2026). Factors associated with frailty status compared to pre-frailty in community-dwelling older adults: A cross-sectional study. Front. Public Health.

[40] Caggiano M., D’ambrosio F., Giordano F., Acerra A., Sammartino P., Iandolo A. (2022). The “Sling” Technique for Horizontal Guided Bone Regeneration: A Retrospective Case Series. Appl. Sci..

[41] Kojima G., Liljas A.E., Iliffe S. (2019). Frailty syndrome: Implications and challenges for health care policy. Risk Manag. Health Policy.

[42] D’ambrosio F., Di Spirito F., De Caro F., Lanza A., Passarella D., Sbordone L. (2022). Adherence to Antibiotic Prescription of Dental Patients: The Other Side of the Antimicrobial Resistance. Healthcare.

[43] Giansanti D., Tiberi Y., Maccioni G. (2008). New wearable system for the step counting based on the codivilla-spring for daily activity monitoring in stroke rehabilitation. Proceeding of the 2008 30th Annual International Conference of the IEEE Engineering in Medicine and Biology Society, Vancouver, Canada, 20–24 August 2008.

[44] Giansanti D., Ricci G., Maccioni G. (2008). Toward the Design of a Wearable System for the Remote Monitoring of Epileptic Crisis. Telemed. E-Health.

[45] Favara G., Barchitta M., Maugeri A., Lio R.M.S., Agodi A. (2024). Sensors for Smoking Detection in Epidemiological Research: Scoping Review. JMIR mHealth uHealth.

[46] Mersha A.G., Bovill M., Eftekhari P., Erku D.A., Gould G.S. (2021). The effectiveness of technology-based interventions for smoking cessation: An umbrella review and quality assessment of systematic reviews. Drug Alcohol. Rev..

[47] Duan L., Zhang S., Yan Q., Chen Y., Su Y., Hu X. (2026). Comparative Efficacy of e-Health Interventions for Quality of Life in Patients with Cancer: A Network Meta-Analysis of Randomized Controlled Trials. Worldviews Evid.-Based Nurs..

[48] Su Y., Zhang S., Duan L., Hu X. (2026). The Effectiveness of Telemedicine-Based Psychosocial Intervention for Fear of Cancer Recurrence, Mindfulness, and Posttraumatic Growth in Cancer Survivors: A Systematic Review and Meta-Analysis of Randomized Controlled Trials. Psycho-Oncol..

[49] Hidalgo I.R., Pisano-González M.M., García C.F., Bywall K.S., Andersson S.W., Avagnina B., Fernández-Feito A. (2026). Digital health literacy and immigrants’ access to digitalized healthcare: A comparative study of Spain, and Sweden—implications for public health policy. BMC Public Health.

[50] Malinovská J., Lustigová M., Koželuhová M., Puchnerová V., Urbanová J., Hloch O., Pálová S., Rozsíval L., Brož J. (2025). Alternativní výrobky pro užívání nikotinu: Současné poznatky. Epidemiol. Mikrobiol. Imunol..

[51] Di Palo M.P., Di Spirito F., Garofano M., Del Sorbo R., Caggiano M., Giordano F., Bartolomeo M., Pessolano C., Giordano M., Amato M. (2025). Effectiveness and Adherence of Standalone Digital Tobacco Cessation Modalities: A Systematic Review of Systematic Reviews. Healthcare.

[52] Coleman C., Ferguson S.G., Nash R. (2024). Barriers to smoking interventions in community healthcare settings: A scoping review. Health Promot. Int..

[53] Ansarian M., Baharlouei Z. (2023). Applications and Challenges of Telemedicine: Privacy-Preservation as a Case Study. Int. J. Infect. Dis..

